# Barriers and facilitators to program directors’ use of the medical education literature: a qualitative study

**DOI:** 10.1186/s12909-022-03104-4

**Published:** 2022-01-19

**Authors:** Asif Doja, Carolina Lavin Venegas, Lindsay Cowley, Lorne Wiesenfeld, Hilary Writer, Chantalle Clarkin

**Affiliations:** 1 Department of Pediatrics, Children’s Hospital of Eastern Ontario, 401 Smyth Rd, Ottawa, ON K1H 8L1 Canada; 2 Children’s Hospital of Eastern Ontario Research Institute, 401 Smyth Rd, Ottawa, ON K1H 8L1 Canada; 3 Department for Innovation in Medical Education Research Support Unit, University of Ottawa, 451 Smyth Rd, Ottawa, ON K1H 8M5 Canada; 4 University of Ottawa, 451 Smyth Rd, Ottawa, ON K1H 8M5 Canada; 5 Department of Virtual Mental Health and Outreach, Centre for Addiction and Mental Health, 1001 Queen St West, Toronto, Ontario M6J 1H4 Canada

**Keywords:** Knowledge translation, Postgraduate training, Medical education literature

## Abstract

**Background:**

It is unclear how often frontline clinical teachers are using this literature and its evidence base in teaching and assessment. Our study purpose was to examine postgraduate program director perspectives on the utilization and integration of evidence-based medical education literature in their teaching and assessment practices.

**Methods:**

The authors conducted semi-structured telephone interviews with a convenience sample of current and former program directors from across Canada. Interviews were transcribed and analyzed inductively to distil pertinent themes.

**Results:**

In 2017, 11 former and current program directors participated in interviews. Major themes uncovered included the desire for time-efficient and easily adaptable teaching and assessment tools. Participants reported insufficient time to examine the medical education literature, and preferred that it be ‘synthesized for them’. (i.e., Best evidence guidelines). Participants recognised continuing professional development and peer to peer sharing as useful means of education about evidence-based tools. Barriers to the integration of the literature in practice included inadequate time, lack of financial compensation for teaching and assessment, and the perception that teaching and assessment of trainees was not valued in academic promotion.

**Discussion:**

Faculty development offices should consider the time constraints of clinical teachers when planning programming on teaching and assessment. To enhance uptake, medical education publications need to consider approaches that best meet the needs of a targeted audiences, including frontline clinical teachers. This may involve novel methods and formats that render evidence and findings from their studies more easily ‘digestible’ by clinical teachers to narrow the knowledge to practice gap.

**Supplementary Information:**

The online version contains supplementary material available at 10.1186/s12909-022-03104-4.

## Background

Clinically relevant medical research can change the practice of clinical medicine through knowledge translation, implementation science, and knowledge mobilization. Knowledge translation and mobilization are dynamic and iterative processes of synthesis and dissemination of research knowledge into practice, while implementation science is the study of these processes [1]. Knowledge translation has a long history of success in transforming clinical research to medical practice [2–5], particularly with regards to evidence-based medicine (defined as the conscientious, explicit, judicious and reasonable use of modern, best evidence in making decisions about the care of individual patients) [6].

In medical education, the corollaries to clinical research and clinical medicine are medical education research and clinical teaching. As such, we can consider evidence-based medical education (or best-evidence medical education) the implementation, by teachers in their practice, of methods and approaches to education based on the best evidence available [2].

Despite considerable growth in medical education publications over time [7], it remains unclear how often frontline clinical teachers use that literature to inform teaching and assessment practices. Concerns have been raised by several medical education scholars regarding a lack of use of medical education evidence by clinician teachers [4] . The underlying factors that sustain this knowledge-to-practice gap remain unclear. As Onyura et al. [4, 8] have discuss ﻿some researchers consider educators to be either poor consumers of education science or lack the skills to understand its research implications [4, 8–10] . Conversely, frontline clinical teachers often maintain that empirical research is often inaccessible or inapplicable to real-world settings [11].

**Fig. 1:**
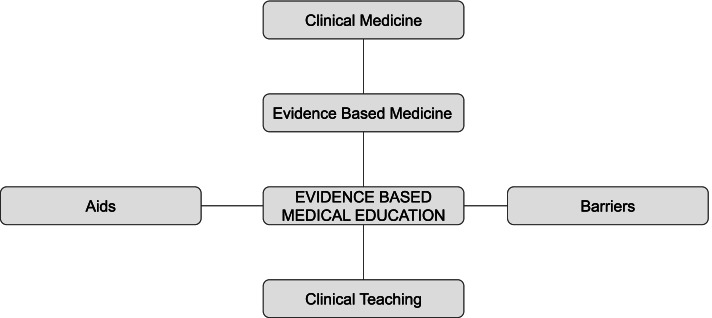
Conceptual Framework for Evidence-Based Medical Education

With a mandate to deliver meaningful teaching and assessment of residents, postgraduate program director (PDs) exemplify frontline clinical teachers and thus should be end users of the medical education literature. The purpose of our study was to examine postgraduate PD perspectives on the utilization and integration of evidence-based medical education tools and literature in their teaching and assessment practices, as well as the barriers and facilitators to their use. The conceptual framework for our study involves the interplay between evidence-based medicine and evidence-based medical education, the relationship between these concepts and clinical medicine / clinical teaching respectively, and the aids and barriers that facilitate or hinder evidence based medical education (see Fig. 1).

## Methods

### Data collection

Using a purposive-snowball sampling approach (i.e. identify & seek volunteers); participants were recruited from a previous national survey of PDs conducted by the authors [11]. At the end of the survey, participants were asked to leave their email address if they wished to be contacted for follow up study.

Review of the pertinent literature and the results of the aforementioned survey informed the development of the interview tool. Interview questions were piloted with two former PDs for completeness, length, and clarity, and the guide was refined based on their feedback (See Supplementary material for the final version of the interview tool).

Semi-structured telephone interviews were conducted with a convenience sample of current and former PDs from across Canada by our research assistant (LC). Interviews continued until saturation was reached and no new themes emerged. Interviews were audio recorded for verbatim transcription. NVivo Version 11 (QSR International Pty Ltd., Melbourne, Australia) was the data management tool used.

### Data analysis

Data collection and analysis followed an iterative process, with recurrent review of data as understandings deepened [12] . Two researchers trained in qualitative methods (CLV and LC) independently reviewed the transcripts. During this process, data were reviewed line-by-line to capture key concepts. The researchers then met to discuss impressions of the data, compare and contrast interpretations within and across transcripts, and develop a shared coding scheme featuring themes and subthemes. They also discussed their underlying assumptions and biases as an awareness raising activity. Data were then coded systematically according to the scheme while allowing for emergence of new previously unseen concepts. The researchers (AD a physician; CLV and LC, non-clinical research assistants; CC a nurse) discussed each transcript to review the coding, resolve conflicting interpretations, revise concepts, and update the coding scheme. Disagreements were discussed among the whole team until consensus was achieved. When coding was complete, the team met to review the findings, clarify interpretations, and refine concepts. They also discussed if any additional lines of inquiry should be explored and confirmed that informational redundancy was met.

### Rigor and trustworthiness of data

*Credibility*, *transferability*, *dependability*, and *confirmability* strategies were enacted to demonstrate trustworthiness of the findings [12] . Peer review, to enact *credibility*, was achieved by presenting the study at a local medical education conference. To enact *transferability*, contextual details and rich description of data were provided, including the context of the study, the participants, and the data collection and analyses processes. *Dependability* of findings was enacted through investigator triangulation (by comparing our findings with the results of our previous survey study), with researchers from both nursing and medical backgrounds with different levels of training performing analytic decision-making and interpretations. To ensure *confirmability* of the study, coding decisions and changes to the coding manual were continuously audited.

This study was approved by the Children’s Hospital of Eastern Ontario Research Institute Ethics Board.

## Results

Eleven former and current PDs participated in interviews (see Table 1 for demographic characteristics of participants); all eleven were respondents from our previous survey. Participants comprised physicians representing various medical and surgical specialties in both adult and pediatric medicine, as well as Radiology and Preventative Health and Public Medicine. Participants represented 6 of Canada’s 13 provinces and territories. Although four participants had conducted medical education research in the past, there were no subjective differences in statements elicited from those who did or did not conduct medical education research. Table 2 list the themes relating to barriers and facilitators to the use of evidence based medical education tools and literature.

**Table 1:** Demographic data of participants, obtained in Ottawa, Ontario Canada, 2017

Demographic Data	*N* = 11
**Gender:**	
Female	5
Male	6
**Years Teaching and Assessing Residents**	
1–5 years	0
6–10 years	4
11–15 years	3
16–20 years	3
21+ years	1
Range:	9–22 years
Mean:	14.5 years
**Years as a Program Director**	
1–5 years	7
6–10 years	4
11–15 years	0
Range:	3–9 years
Mean:	5.7 years
**Conducted Medical Education Research in the Past?**	
Yes	4
No	7

**Table 2:** Themes relating to barriers and facilitators to the use of the medical education literature, obtained in Ottawa, Ontario, 2017

**Access to information**	
**Modifiable/Practical Tools**	
**Advantages of Shared Resources**	
**Faculty Attitudes**	
**Time as a Barrier**	
**Value of Teacher Role**	
**Education/Training in Medical Education Theory**	

### Access to information

PDs found the sheer amount of educational evidence available daunting. As a result, when they had time to examine the medical education literature, they chose easily accessible resources and tools in terms of language, length, and clinical application. Instead of examining the primary literature, PDs preferred reviews, summaries and guidelines which synthesized key concepts in medical education, such as the Best Evidence in Medical Education (BEME) series [4].

*“I think that there’s a lot of great education research going on but I feel that there’s a big gap in translation to all of the … program directors. So a similar example would be in the clinical world, there’s … tons of primary research being done, but if the research is being done and nobody’s reading it, it doesn’t really matter”* [(Program Director (PD) 1]

### Modifiable/practical tools

PDs stated that they were looking for teaching strategies and assessment tools that were easy to implement, practical, and helpful in their day-to-day educational activities. Ideally, they were seeking strategies and tools that were easy to learn and required minimal training. Additionally, they sought tools that could be modified with minimal effort and adapted to diverse learning environments, training specialties and even learner levels.

### Advantages of shared resources

Participants desired the strategic dissemination of educational strategies, innovations and research to alleviate the need for them to review the educational literature. Participants suggested a variety of ways to accomplish this, including engagement in academic conferences, seminars, peer discussions and continuing medical education workshops. They also preferred not to ‘re-invent the wheel’ with regards to teaching and assessment strategies and felt that local centralized repositories would be a welcome method to facilitate sharing.

*“Rather than me kind of going and looking at a literature myself and analyzing what works or what doesn’t work, to have someone kind of do that legwork for me and to demonstrate that it can be very helpful”*(PD8)

### Faculty attitudes

Local practice contexts were felt to be both a facilitator and a barrier to the use of the medical education research. For example, local medical education ‘champions’ were believed to promote co-learning and the uptake of educational tools and interventions in practice. Conversely, a local culture of resistance to change was perceived as a significant barrier to knowledge mobilization. Several participants reported that their colleagues were often wary of changing the traditional ways of teaching and assessment.

*“So I can think this is the best thing since sliced bread, but if I can’t get my, you know, faculty on board to actually use my assessment tool or to teach or to do those sort of pieces, it just doesn’t go anywhere”* (PD6)

### Time as a barrier

A consistent issue heard from PDs was the lack of time to adequately peruse, read, interpret and implement findings from the medical education literature. Participants expressed that although, as clinical teachers, they felt a need to keep abreast of developments in medical education, there were too many competing demands -- including clinical and administrative duties -- for this to be sustainable.

*“One of these barriers would be time … we have various limited protected time and so having more time to successfully research what are useful validated tools that could relate to our program would be helpful.”(PD9)*

### Education/training in medical education theory

Many PDs expressed a desire to strengthen their knowledge base regarding educational theory, learning, and assessment in medical education. Several PDs noted that they were thrust into leadership roles in medical education without sufficient background knowledge. This perceived knowledge gap was cited as a barrier to the synthesis and deeper understanding of medical education literature.

*“Medical education uses its own vocabulary and I’m not always sure that I understand what people are talking about when they use the vocabulary. So I guess it’s the epistemology of medical education isn’t that clear to me since I haven’t had any professional training in it … sometimes I’m not actually sure what it is that I’m even looking for”*(PD7)

*“I think there is a big disconnect between medical education research and medical education practice and you’ve got a lot more people who have to do the teaching and assessment and people who do research. So I mean obviously you're looking at the disconnect between the two. And sometimes when I read the medical education literature, I kind of go, “That’s not very helpful”*(PD2)

## Discussion

Participants in our study identified several barriers and facilitators to their access and application of the medical education literature. Onyura et al. [4] examined this issue at a local level with undergraduate medical education clinical teachers at single institution and found that faculty only occasionally engaged with medical education research knowledge. Similar to our study, time was a major constraining factor. This has been noted previously, with some estimating it would take 75 min per working day for a physician to remain current with the medical education literature [4]; this amount of time is untenable for most busy clinicians.

Onyura et al. [4] also identified resistance to change from faculty as a perceived barrier; as participants in our study discussed, one way to address this would be to have local physician “champions” to help shepherd in new medical education initiatives. They also found that participants were more likely to use a particular tool if it was presented at a faculty development program, if it was locally developed, or if it was recommended by a peer, similar to what was shown in our study. However, as has been discussed by others [13], local change needs to occur at more than just the individual level; for true change to occur, it must occur at the organizational level. As such, if organizations wish to initiate new medical education initiatives, they may choose to tools such as the Specialty training’s Organizational Readiness for curriculum Change (STORC) [14] or the Medical School’s Organizational Readiness for Curriculum Change (MORC) [15, 16]. PD’s have the potential to help facilitate organization change due to the dual lens they carry, as frontline clinical teachers as well as medical education leaders.

Thomas et al. [17] surveyed 396 members of the Association for Medical Education in Europe (AMEE) and found that 83% of respondents used research to inform their educational practices. However, 59% felt that (other) educators in the health professions made little use of research findings. Many of the respondents in Thomas et al’s survey might reasonably be expected to classify themselves as medical education researchers, thereby possessing detailed knowledge of medical education research. In contrast, only 4 of our 11 respondents in our study participated in medical education research. As such, our sample may have been more representative of clinician teachers as opposed to those who conduct medical education research.

When looking at the literature on barriers to evidence based medicine (EBM) in general, in a systematic review of barriers to the use of EBM by general practitioners [18] demonstrated numerous factors felt to be responsible for decreased uptake of EBM. For example, barriers related to the actual evidence included a perceived lack of evidence and perceived poor quality of evidence Some felt the available evidence is contradictory, not up to date, and liable to time delays. Others were skeptical of evidence derived from certain sources that may be biased by, i.e. the pharmaceutical industry [18]. Similar to our study, some felt intimidated by the overall quantity of evidence and the lack of time to review it. In addition to time, some found they had limited access to the evidence, sometimes from computer/internet problems. Many cited a lack of skills in using EBM, similar to our study where respondents had difficulty understanding the concepts and theories in medical education [18]. Colleagues use of EBM also tended to influence use, similar to what we noted.

Unlike our study, patient preferences sometimes play a role in EBM, which is less of a factor in medical education. For example, when the evidence-based preferences of the physicians and the wishes of the patient do not match, the physician may feel a barrier to convincing the patient to proceed with a given course of treatment [18] . Finally, some individuals felt that institutional support was often lacking to support EBM, similar to our findings in the medical education realm.

Another systematic review of the barriers to EBM in clinical medicine was conducted by ﻿﻿Sadeghi-Bazargani et al. [16]. Again, a lack of resources, lack of time, inadequate skills, and inadequate access, lack of knowledge and financial barriers were found to be the most common barriers to EBM, echoing many of the findings in our study.

A strength of our study is the inclusion of participants from institutions across Canada. The PDs in this study representing a breadth of specialties, provide education to diverse learners, and are involved in the care of heterogeneous patient populations. As our study mainly focussed on frontline clinical teachers; future studies could conceivably examine medical education to explore whether similar barriers and facilitators to the utilization of the medical education research are present across groups. Future studies examining barriers and facilitators to the mobilization of knowledge using an interdisciplinary lens could also provide useful insights to medical education.

## Conclusions

Our national qualitative study of program directors across Canada, elaborates on several barriers and facilitators to the use of the medical education literature by frontline clinician teachers and leaders. Particularly, we found that frontline clinical teachers require more protected time to review the medical education literature and that they prefer to have the evidence summarized in a practical, easy to use manner. They feel that aids to the use of the medical education literature include local champions and the use of shared resources. Finally, they expressed concern over the need to better understand educational theory, as it relates to learning and assessment. These findings have relevance to both medical education researchers, faculties of medicine, medical education publications and medical education conferences.

### Recommendations for medical education researchers

There have been repeated calls in the medical education literature for investigators to demonstrate that their research offers solutions to real world problems [3]. As Archer et al. state, “medical education researchers need to properly communicate the importance of their research, translating its findings for the medical profession, the wider healthcare community, the academic community, research commissioners, and the public”. While this may be true, when comparing clinical research to medical education, it is important to emphasize that key differences exist between these types of research. Greenhalgh et al. [3] argue that ﻿educational research questions, in contrast to clinical research questions, have a more complex taxonomy, a less direct link with study design, and no universally accepted criteria for assessing validity. Van der Vleuten and Dreissen [19] discuss that ﻿evidence in education is more than insights from empirical findings stemming from educational research, and includes theories that are derived from the empirical evidence.

Overall, based on our findings, as well as the suggestions of various medical education scholars, we suggest medical education researchers should consider the following when conducting research:

- When discussing theory, ensure that readers know the practical applications of theories presented. Kaufman [20] argues that medical education theory should not be relegated to the “ivory tower” and has provided examples in the past on how educational theories can be linked to practice [21–23] .
- Medical education researchers should contribute to education evidence that explains why educational strategies work and under which conditions [24].

### Recommendations for faculties of medicine, medical education publications and medical education conferences

Our findings also provide a basis for recommendations for Faculties of Medicine, medical education publications and medical education conferences as follows:

- Faculty development programs should consider the time constraints of clinical teachers when disseminating information on teaching and assessment tools. They should use tools that are simple, easy to use, and widely adaptable
- Faculties of Medicine should focus on increased ‘protected” time for program directors and clinical teachers
- Medical education publications should consider approaches that best meet the needs of their target audiences. This may involve novel methods and formats that render evidence and findings from their studies more easily ‘digestible’ i.e. infographics, social media, video briefs, podcasts, lay language summaries, etc.
- Medical education conferences and continuing medical education initiatives should ensure they meet the needs of learners at all levels (Novice, Intermediate, Advanced)
- Integrated, peer-to-peer co-learning and mentorship opportunities could potentially enhance the uptake of research in practice. These could include locally-hosted or virtual ongoing learning opportunities such a journal clubs, seminars, and the identification of medical education champions.
- Healthcare organizations need to foster supportive learning environments that attract clinical teachers with medical education training and promote ongoing continuing professional development

## Supplementary Information

**Additional file 1.**

## Data Availability

Not Applicable.
