# Supplementary material for: Barriers and facilitators to program directors’ use of the medical education literature: a qualitative study
Source: BMC Med Educ. 2022 Jan 19;22:45. doi: 10.1186/s12909-022-03104-4 (PMC8772128; doi:10.1186/s12909-022-03104-4)
Supplement: Supplementary file 1 — Additional file 1. [file 12909_2022_3104_MOESM1_ESM.doc]

**Supplementary Material**

**Interview Guide**

Aids and barriers to the use of existing tools and the medical education literature in teaching the CanMEDS roles: A mixed-methods study

DATE: _______________________________

TIME: _______________________________

PARTICIPANT ID: _______________________________

INTERVIEWER: _______________________________

First I want to give you a bit of background information on this study:

Despite the existence of strategies and tools to aid in the teaching and assessment of CanMEDS roles, clinical educators believe they are insufficiently informed about how to teach and assess these roles.

In the survey phase of this study—that you previously participated in-- we aimed to establish what strategies and tools are used in the teaching and assessment of these roles (for example, toolkits provided by local, provincial or national organizations) and examine the extent to which they are used.

We also aimed to examine the extent to which the medical education literature (for example research articles in peer reviewed journals) was incorporated into these educational practices.

We now want to understand the potential aids and barriers to such use.

To begin, I would like to ask you a few questions about yourself to provide some context for the interview.

Specialty/subspecialty: _______________________________

Number of years teaching/assessing **medical residents** (total, including residency):___________

Number of years as a Program Director: _______________________________

Conduct medical education research: Yes/No _______________________________

Now we are going to get into the focus of the interview. The questions are grouped in two sections: A and B. First, in section A, I am going to ask you questions about what **helps** you use evidence-based **teaching strategies** and **assessment tools** for teaching and assessing the CanMEDS roles. I will then ask about the **barriers or challenges** to using those strategies and tools.

In section B, I will ask you to reflect on your use of medical education literature when you teach and assess the CanMEDS roles. As always, there are no right or wrong answers. Let’s get started:

1. **Aids to utilizing evidence-based teaching strategies and assessment tools**

First we are going to talk about evidence-based **teaching strategies**:

1. In your opinion, what **helps** you to be able to use evidence-based **teaching strategies for teaching** the CanMEDS roles? *[Examples that might arise are willingness to use, valuing, and access]*

*Prompt [if needed]:* Can you further explain **why** this is helpful? Please illustrate with an example.

The next question focuses on evidence-based **assessment tools:**

1. In your opinion, what **helps** you to be able to use evidence-based assessment tools **for assessing** the CanMEDS roles? *[Examples that might arise are willingness to use, valuing, and access]*

*Prompt [if needed]:* Can you further explain **why** this is helpful? Please illustrate with an example.

1. How do you believe these **helpful** things, related to using evidence-based teaching strategies and assessment tools, can be supported by people/departments/institutions?

**Barriers to utilizing evidence-based teaching strategies and assessment tools**

In this question we are interested in **evidence-based teaching strategies:**

1. In your opinion, what **hinders** the use of evidence-based **teaching strategies for teaching** the CanMEDS roles? *[Examples that might arise are time and ability to evaluate quality]*

*Prompt [if needed]:* Can you further explain **why** this is hindering? Please illustrate with an example.

This question focuses on **evidence-based assessment tools:**

1. In your opinion, what **hinders** the use of evidence-based assessment tools **for assessing** the CanMEDS roles? *[Examples that might arise are time and ability to evaluate quality]*

*Prompt [if needed]:* Can you further explain **why** this is hindering? Please illustrate with an example.

1. How do you believe these **hindering** things, related to using evidence-based teaching strategies and assessment tools, can be overcome by people/departments/institutions?
2. **Use of the medical education literature to inform the teaching and assessment of the CanMEDS roles**
3. How does the medical education literature inform your teaching of the CanMEDS roles? *[Follow with questions 8 and 9 if participant uses the literature or question 10 if does not]*
4. *If uses the medical education literature:* In your opinion, what helps in using the medical education literature for informing the teaching of the CanMEDS roles?
5. *If uses the medical education literature:* In your opinion, what hinders the use of the medical education literature for informing the teaching of the CanMEDS roles?
6. *If does not use the medical education literature:* Can you explain **why** the medical education literature does not inform your teaching of the CanMEDS roles?
7. How does the medical education literature inform your assessment of the CanMEDS roles? *[Follow with questions 12 and 13 if participant uses the literature or question 14 if does not]*
8. *If uses the medical education literature:* In your opinion, what helps in using the medical education literature for informing the assessment of the CanMEDS roles?
9. *If uses the medical education literature:* In your opinion, what hinders the use of the medical education literature for informing the assessment of the CanMEDS roles?
10. *If does not use the medical education literature:* Can you explain **why** the medical education literature does not inform your assessment of the CanMEDS roles?
11. Is there anything you would like to add to the interview about the use of the medical education literature and evidence-based teaching strategies and assessment tools for teaching and assessing the CanMEDS roles?
